# MIR503HG impeded ovarian cancer progression by interacting with SPI1 and preventing TMEFF1 transcription

**DOI:** 10.18632/aging.204147

**Published:** 2022-06-28

**Authors:** Jun Tian, Lei Yang, Zhongtai Wang, Haiya Yan

**Affiliations:** 1 Department Gynecology, HuaiHe Hospital of HeNan University, Kaifeng, HeNan, China; 2 Department Gynecology, Baoji Maternal and Child Health Care Hospital, Baoji, Shaanxi, China; 3 Health Management Center, Taizhou Hospital of Zhejiang Province, Taizhou, Zhejiang, China

**Keywords:** MIR503HG, SPI1, TMEFF1, ovarian cancer

## Abstract

MIR503 host gene (MIR503HG) acts as an important tumor suppressor in many human cancers, but its role and regulatory mechanism in ovarian cancer need to be further studied. In this study, lower expressed MIR503HG was observed in ovarian tumor tissues and cells than in adjacent normal tissues and normal human ovarian epithelial cells. MIR503HG overexpression impaired the proliferative, invasive and EMT properties, and facilitated cell apoptosis in ovarian cancer cells. Nuclear and cytoplasmic separation test suggested that MIR503HG was mainly expressed in the nucleus. RNA immunoprecipitation and RNA pull-down assays confirmed that MIR503HG could bind to transcription factor SPI1 (Spi-1 proto-oncogene), and dual luciferase reporter gene and Chromatin immunoprecipitation assays verified that SPI1 could bind to TMEFF1 (Transmembrane protein with EGF like and two follistatin like domains 1) promoter, suggesting that MIR503HG suppressed TMEFF1 expression by competitively binding SPI1 and blocking transcriptional activation of TMEFF1. Moreover, interference with TMEFF1 reversed the promotion effect of MIR503HG silence on the malignant behaviors of ovarian cancer cells. Moreover, MIR503HG knockdown activated the MAPK and PI3K/AKT pathways by increasing the expression of TMEFF1. In addition, overexpression of MIR503HG *in vivo* suppressed the tumorigenic ability in nude mice. In conclusion, MIR503HG acted as a tumor suppressor lncRNA in ovarian cancer by suppressing transcription factor SPI1-mediated transcriptional activation of TMEFF1.

## INTRODUCTION

Ovarian cancer is one of the deadliest types of cancer affecting the female reproductive tract and the most common cause of cancer death in women [1, 2]. Despite advances in treatments that include surgery, chemotherapy and radiation, the five-year survival rate for ovarian cancer patients is still only 30% [3]. An important cause is the asymptomatic development of early-stage ovarian cancer, which leads to patients being diagnosed at a later stage of the disease with metastasis [4]. In addition, ovarian cancer has high recurrence rate and metastatic potential [5]. Therefore, there is an urgent need to explore new diagnostic or prognostic biomarkers and therapeutic targets.

Current researches have demonstrated that lncRNAs play important roles in transcription, genome imprinting, mRNA degradation, protein dynamics, and as RNA decoy or scaffold [6, 7]. Increasing evidence suggested that lncRNAs are involved in a series of biological functions such as cell proliferation, apoptosis, invasion and tumor metastasis of ovarian cancer [1, 8], and many lncRNAs that can be used as new biomarkers of ovarian cancer are identified by lncRNA microarray spectrum [9]. LncRNA DANCR regulates the expression of vascular endothelial growth factor A by adsorbing miR-145 to promote ovarian cancer tumor angiogenesis [5]. By targeting RhoC, lncRNA ABHD11-AS1 promotes proliferation and invasion and inhibits apoptosis of ovarian cancer cells *in vitro*, and contributes to tumor growth and metastasis in mice [10]. MIR503 host gene (MIR503HG) is a 786 bp long lncRNA located on chromosome Xq26.3 [11]. It acts as an important tumor suppressor in various human cancers. MIR503HG is down-regulated in ovarian cancer tissues, and its low expression predicts poor survival of ovarian cancer patients.

Recent studies showed that lncRNAs can interact with transcription factors to regulate the transcription of downstream genes. Transcription factors was reported to promote tumorigenesis by activating oncogenes or inhibiting tumor suppressor genes [12]. The transcription factor SPI-1 (Spi-1 proto-oncogene) can participate in cancer progression by regulating the transcription of oncogenes [13]. Moreover, SPI1 promotes lncRNA SNHG6 transcription by binding to the SNHG6 promoter, thereby promoting the migration and invasion of non-small cell lung cancer (NSCLC) cells [14]. Transmembrane protein with EGF like and two follistatin like domains 1 (TMEFF1) is a member of the CTA family (CT 120-1), and it is a transmembrane protein. TMEFF1 is initially reported to be differentially expressed in brain tumor tissues and normal brain tissues, and played a tumor suppressor role in brain cancer [15]. TMEFF1 is subsequently found to be expressed in breast and colon cancer cell lines [16, 17]. A recent research indicated that TMEFF1 is highly expressed in ovarian cancer tissues and cell lines, and its high expression predicts a shorter overall survival period for patients with ovarian cancer. In addition, overexpression of TMEFF1 promotes the proliferation, migration and invasion of ovarian cancer cells, and inhibits cell apoptosis [18].

In this study, we found that overexpression of MIR503HG suppressed ovarian cancer cell proliferation and invasion, and contributed to cell apoptosis. Mechanism researched showed that MIR503HG affected the transcriptional regulation effect of SPI1 on the downstream gene TMEFF1 by interacting with the SPI1, thereby suppressing the tumorigenicity of ovarian cancer cells.

## RESULTS

### MIR503HG expression was down-regulated in ovarian cancer cells and tissues

**Figure 1:**
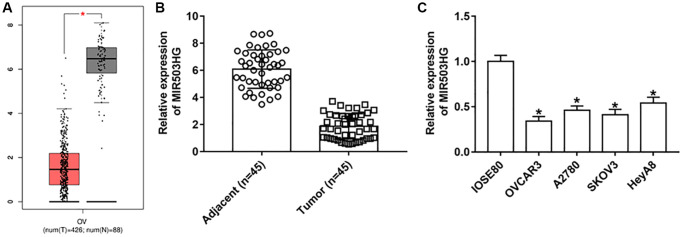
**MIR503HG expression was down-regulated in ovarian cancer cells and tissues.** (**A**) GEPIA online database was used to analyze the expression of MIR503HG in ovarian cancer tissues and adjacent normal tissues. (**B**) MIR503HG expression in 45 paired ovarian cancer tissues (*n* = 45) and adjacent normal tissues (*n* = 45) was measured with RT-qPCR. (**C**) MIR503HG expression in ovarian cancer cell lines (OVCAR3, A2780, SKOV3, and HeyA8) and normal human ovarian epithelial cells (IOSE80) was detected. ^*^P < 0.05. Each test was repeated at least three times independently.

GEPIA online database was used to analyze the expression of MIR503HG in ovarian cancer tissues and adjacent normal tissues, and lower expressed MIR503HG was observed in ovarian tumor tissues than in non-cancerous tissues (Figure 1A). Moreover, RT-qPCR results confirmed that MIR503HG expression was downregulated in collected ovarian cancer tissues and cell lines (OVCAR3, A2780, SKOV3, and HeyA8), compared with that in adjacent normal tissues and normal human ovarian epithelial cells (IOSE80) (Figure 1B, 1C).

### MIR503HG overexpression alleviated malignant behaviors of ovarian cancer cells

**Figure 2:**
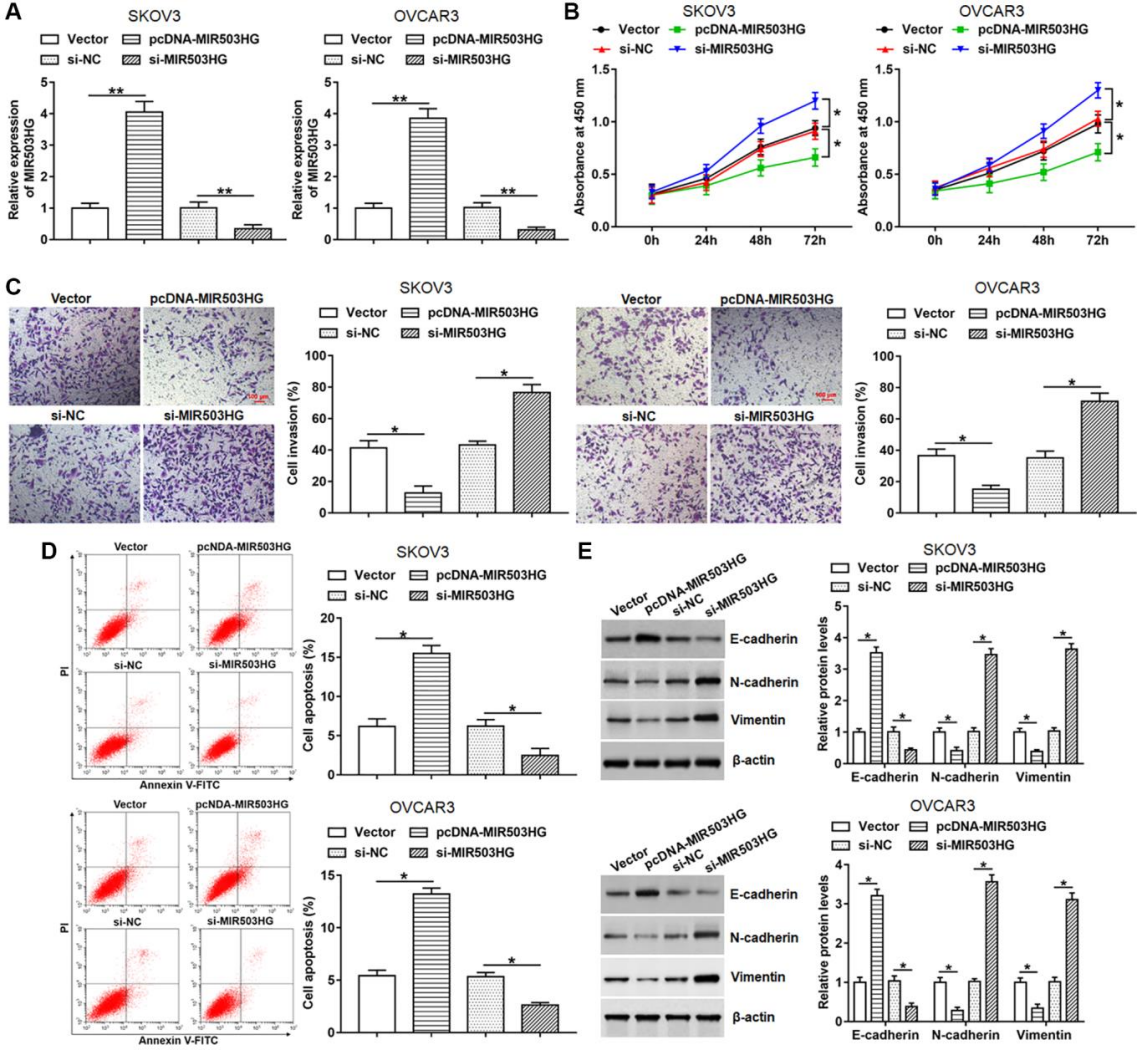
**MIR503HG overexpression alleviated malignant behaviors of ovarian cancer cells.** pcDNA-MIR503HG, MIR503HG siRNA and respective controls were transfected into SKOV3 and OVCAR3 cells, after transfection for 48 h, (**A**) MIR503HG expression was measured with RT-qPCR. (**B**) CCK-8 was performed to detect cell proliferation. (**C**) Cell invasion was determined with Transwell assay. (**D**) Flow cytometry was used to analyze cell apoptosis. (**E**) The protein levels of E-cadherin, N-cadherin and Vimentin were assessed with Western blotting. ^*^P < 0.05, ^**^P < 0.01. *n* = 6 in each group. Each test was repeated at least three times independently.

Next, pcDNA-MIR503HG, MIR503HG siRNA or respective controls were transfected into SKOV3 and OVCAR3 cells to evaluate the effect of MIR503HG on cell behaviors. Upregulated expression of MIR503HG was detected after transfection with pcDNA-MIR503HG, and reduced expression of MIR503HG was observed after interference with MIR503HG (Figure 2A). Moreover, our data displayed that overexpression of MIR503HG markedly suppressed cell proliferation and invasion, and facilitated cell apoptosis, while MIR503HG silence played an adverse role (Figure 2B–2D). Furthermore, we found that enforced expression of MIR503HG notably increased the expression of E-cadherin and reduced the expression of N-cadherin and Vimentin, and MIR503HG knockdown showed an opposite effect (Figure 2E).

To explore whether overexpression of MIR503HG regulates cell behaviors by promoting miR-503 expression, SKOV3 cells were transfected with pcDNA-MIR503HG alone or together with miR-503 inhibitor. The data showed that transfection with pcDNA-MIR503HG upregulated MIR503HG and miR-503 expression, and treatment with miR-503 inhibitor suppressed miR-503 expression without changing MIR503HG expression (Supplementary Figure 1A). In addition, overexpression of MIR503HG restrained cell proliferation and invasion, and promoted cell apoptosis, while interference with miR-503 did not change the effect of MIR503HG on cell proliferation, invasion and apoptosis (Supplementary Figure 1B–1D). These data suggested that MIR503HG functioned independently of miR-503.

### MIR503HG interacted with SPI1

**Figure 3:**
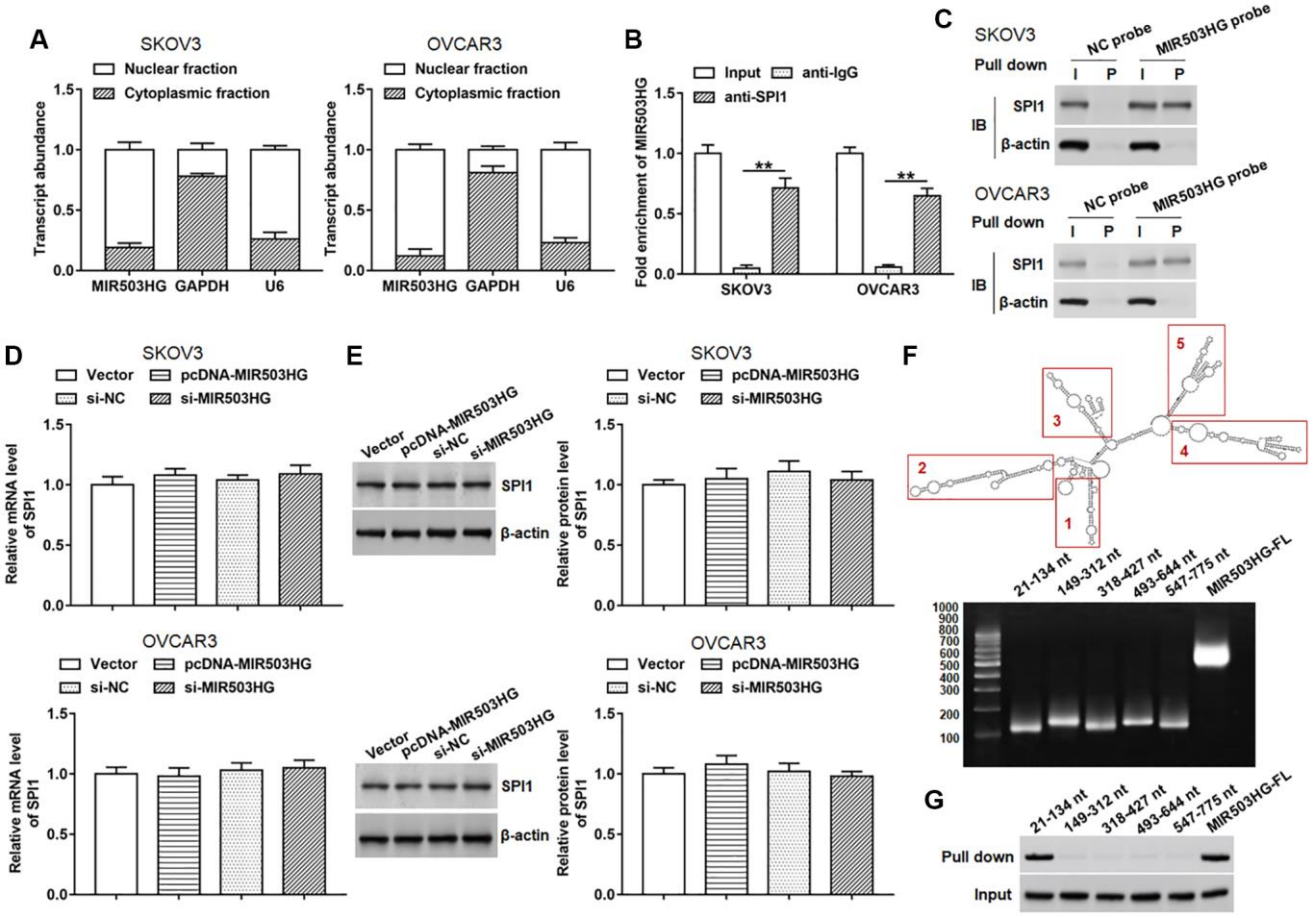
**MIR503HG interacted with SPI1.** (**A**) MIR503HG expression in cytoplasm and nucleus was evaluated in SKOV3 and OVCAR3 cells. U6 was used as a nuclear marker, and GAPDH was used as a cytoplasmic marker. (**B**) RIP assay was conducted with SPI1 antibody in SKOV3 and OVCAR3 cells, and anti-IgG was used as an internal control. (**C**) RNA pull-down experiment was performed by using biotinylated MIR503HG transcripts. (**D**, **E**) SPI1 mRNA and protein levels were determined after transfection with pcDNA-MIR503HG, MIR503HG siRNA or respective controls. (**F**) RNAfold web server was used to predict the secondary structure of MIR503HG, and MIR503HG sequence was divided into 5 truncated parts according to its secondary structure. (**G**) RNA pull-down assay was carried out with biotinylated MIR503HG truncated fragments. ^**^P < 0.01. *n* = 6 in each group. Each test was repeated at least three times independently.

Since the subcellular localization of lncRNA is closely related to its function, we first performed nucleo-cytoplasmic separation test and found that MIR503HG was mainly located in the nucleus in SKOV3 and OVCAR3 cells (Figure 3A). lncMAP (http://bio-bigdata.hrbmu.edu.cn/LncMAP/) was applied to predict potential MIR503HG binding transcription factors. Among these transcription factors, we noticed that SPI1 was reported to promote malignant progression of ovarian cancer. Whereafter, RIP and RNA pull-down assays were performed to verify the binding of MIR503HG and SPI1. The results of RIP showed that MIR503HG was prominently enriched in immunocomplex precipitated by anti-SPI1, compared with that in immunocomplex precipitated by anti-IgG (Figure 3B). Moreover, compared with using NC probe, SPI1 protein was significantly enriched in RNA-protein immune complexes pulled down by using MIR503HG probe (Figure 3C). We also explored the regulatory effect of MIR503HG on the expression of SPI1 and found that neither overexpression nor interference with MIR503HG did not alter the mRNA and protein expression levels of SPI1 (Figure 3D, 3E). Furthermore, we divided MIR503HG sequence into five truncated parts according to the stem-loop structure of MIR503HG to explore the binding region of SPI1 on the MIR503HG sequence. The RNA pull-down assay revealed that SPI1 mainly bound to MIR503HG fragment 21-134 nt (Figure 3F, 3G).

### MIR503HG restrained SPI1-mediated transcriptional activation of TMEFF1

**Figure 4:**
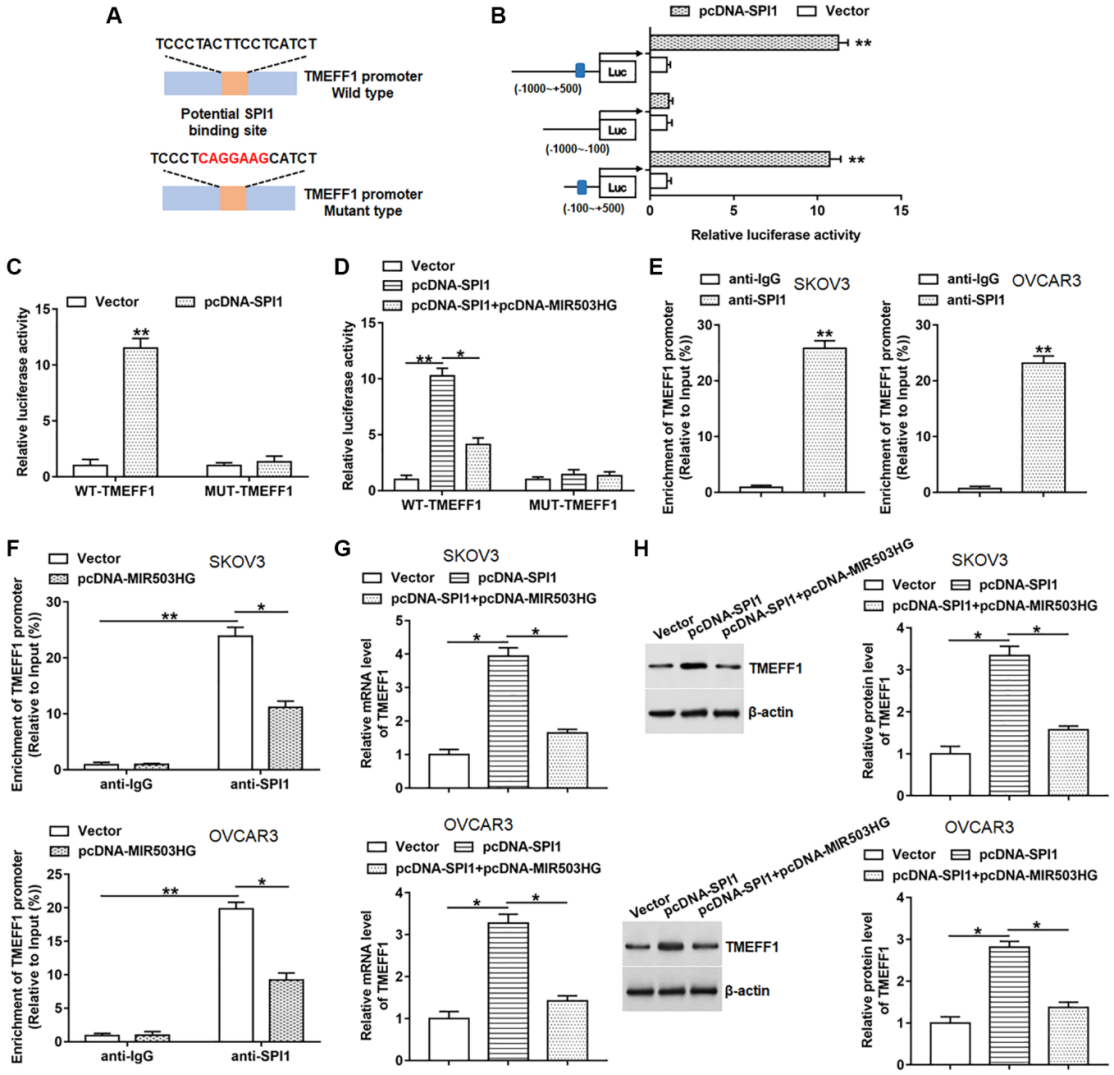
**MIR503HG restrained SPI1-mediated transcriptional activation of TMEFF1.** (**A**) ChIPBase database was used to predict the binding motif of SPI1 in TMEFF1 promoter region, and the mutation sites were shown in red. (**B**) Double luciferase reporter gene assay was performed using different truncated sequences of TMEFF1 promoter region. (**C**) WT-TMEFF1/MUT-TMEFF1 reporters were transfected into HEK293T cells together with pcDNA-SPI1/control vector, and the relative luciferase activities were detected. (**D**) HEK293T cells were transfected with WT-TMEFF1/MUT-TMEFF1 reporters together with pcDNA-SPI1 or pcDNA-SPI1 + pcDNA-MIR503HG, and the relative luciferase activities were analyzed. (**E**) ChIP assay was carried out to verify the binding of SPI1 to the TMEFF1 promoter region in SKOV3 and OVCAR3 cells. (**F**) ChIP assay was conducted with anti-SPI1 or anti-IgG in SKOV3 and OVCAR3 cells transfected with control vector or pcDNA-MIR503HG. (**G**, **H**) pcDNA-SPI1 was transfected into cells alone or together with pcDNA-MIR503HG, after transfection for 48 h, the mRNA and protein levels of TMEFF1 were measured. ^*^P < 0.05, ^**^P < 0.01. *n* = 6 in each group. Each test was repeated at least three times independently.

By using the ChIPBase database, we found that SPI1 might bind to the promoter region of TMEFF1, and the binding motif is “TCCCTACTTCCTCATCT” (Figure 4A). Next, we constructed three truncated vectors of the TMEFF1 promoter part to explore the binding region of SPI1. The results showed that the luciferase activities of the reporters containing the predicted binding site and full-length sequence were markedly upregulated after overexpression of SPI1. While SPI1 overexpression did not change the luciferase activity of the reporter without predicted binding site (Figure 4B). Moreover, we constructed two luciferase reporters consisting of wild-type (WT) and mutant-type (MUT) TMEFF1 promoters, and observed that overexpression of SPI1 significantly promoted the luciferase activities of the WT-TMEFF1 reporter without affecting the luciferase activities of the MUT-TMEFF1 reporter (Figure 4C). Whereas overexpression of MIR503HG notably suppressed the promotion effect of SPI1 on the luciferase activities of the WT-TMEFF1 reporter, but did not change the luciferase activities of the MUT-TMEFF1 reporter (Figure 4D). Moreover, ChIP assay was carried out to verify the binding of SPI1 to the TMEFF1 promoter region. The data displayed that TMEFF1 promoter was markedly enriched in the SPI1 antibody-precipitated complex, and transfection with pcNDA-MIR503HG significantly suppressed the binding of SPI1 to the TMEFF1 promoter region (Figure 4E, 4F). Furthermore, overexpression of SPI1 increased the mRNA and protein levels of TMEFF1 in SKOV3 and OVCAR3 cells, which was reversed by transfection with pcNDA-MIR503HG (Figure 4G, 4H).

### Silencing TMEFF1 reversed the promotion effect of MIR503HG knockdown on malignant behaviors of ovarian cancer cells

**Figure 5:**
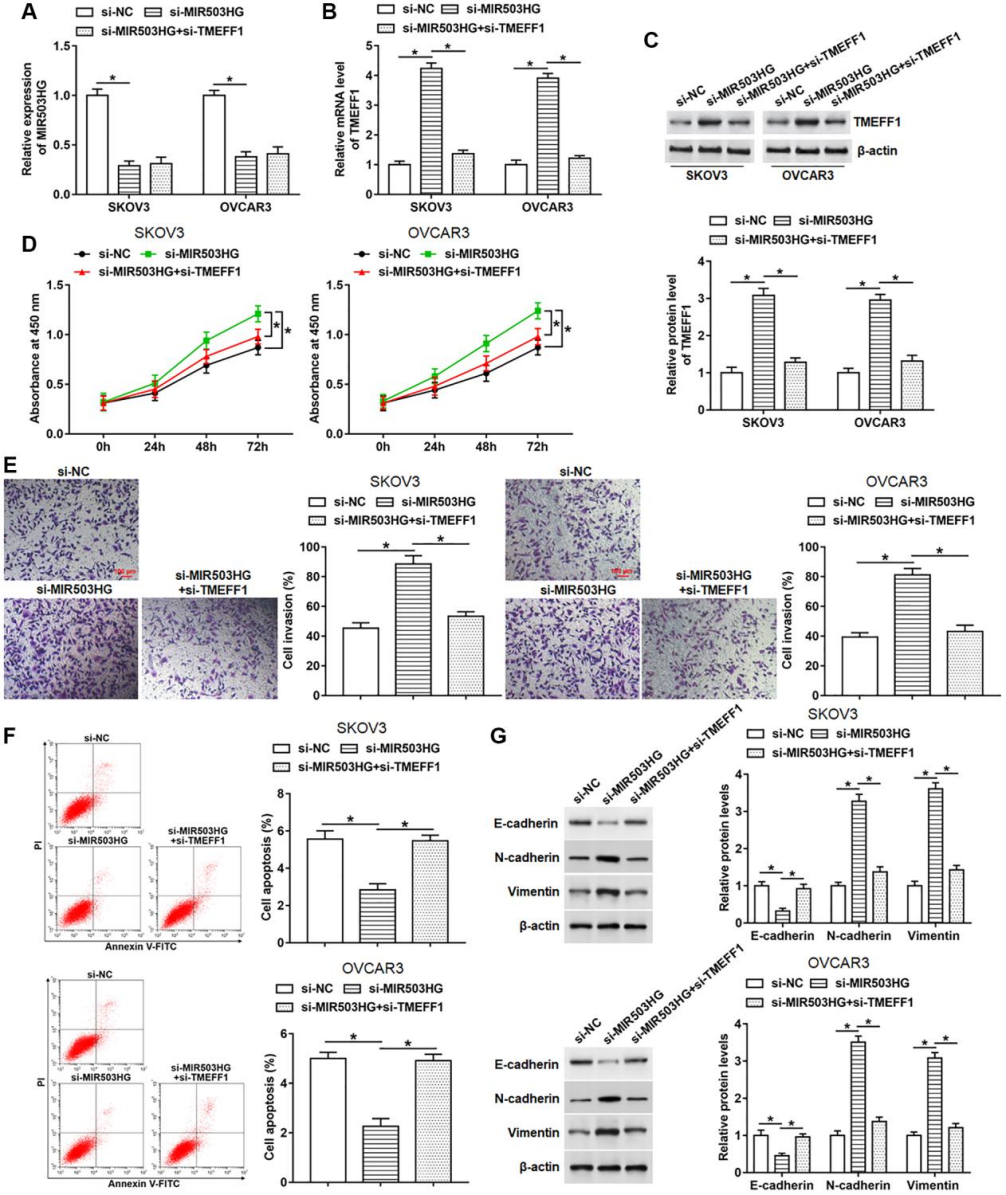
**Silencing TMEFF1 reversed the promotion effect of MIR503HG knockdown on malignant behaviors of ovarian cancer cells.** MIR503HG siRNA was transfected into SKOV3 and OVCAR3 cells alone or together with TMEFF1 siRNA, after transfection for 48 h, (**A**) the expression of MIR503HG was measured. (**B**, **C**) The mRNA and protein levels of TMEFF1 were detected with RT-qPCR and Western blotting. (**D**, **E**) CCK-8 and Transwell assays were performed to measure cell proliferation and invasion. (**F**) Flow cytometry was carried out to evaluate cell apoptosis. (**G**) The expression of E-cadherin, N-cadherin and Vimentin was analyzed. ^*^P < 0.05. *n* = 6 in each group. Each test was repeated at least three times independently.

To explore whether MIR503HG affected cell behaviors by regulating TMEFF1 expression, SKOV3 and OVCAR3 cells were transfected with MIR503HG siRNA alone or together with TMEFF1 siRNA. The results of RT-qPCR and Western blotting assays indicated that transfection with MIR503HG siRNA notably inhibited MIR503HG expression and upregulated TMEFF1 mRNA and protein levels. And compared with MIR503HG siRNA treatment group, the co-transfection of MIR503HG siRNA and TMEFF1 siRNA suppressed the mRNA and protein expression of TMEFF1 without altering the expression of MIR503HG (Figure 5A–5C). Moreover, TMEFF1 knockdown reversed the upregulation of cell proliferation and invasion, and the decrease of cell apoptosis caused by interference with MIR503HG (Figure 5D–5F). Additionally, MIR503HG knockdown observably inhibited E-cadherin expression and increased N-cadherin and Vimentin expression, which was suppressed by silencing TMEFF1 (Figure 5G).

### Silencing MIR503HG activated MAPK and PI3K/AKT pathways by increasing TMEFF1 expression

**Figure 6:**
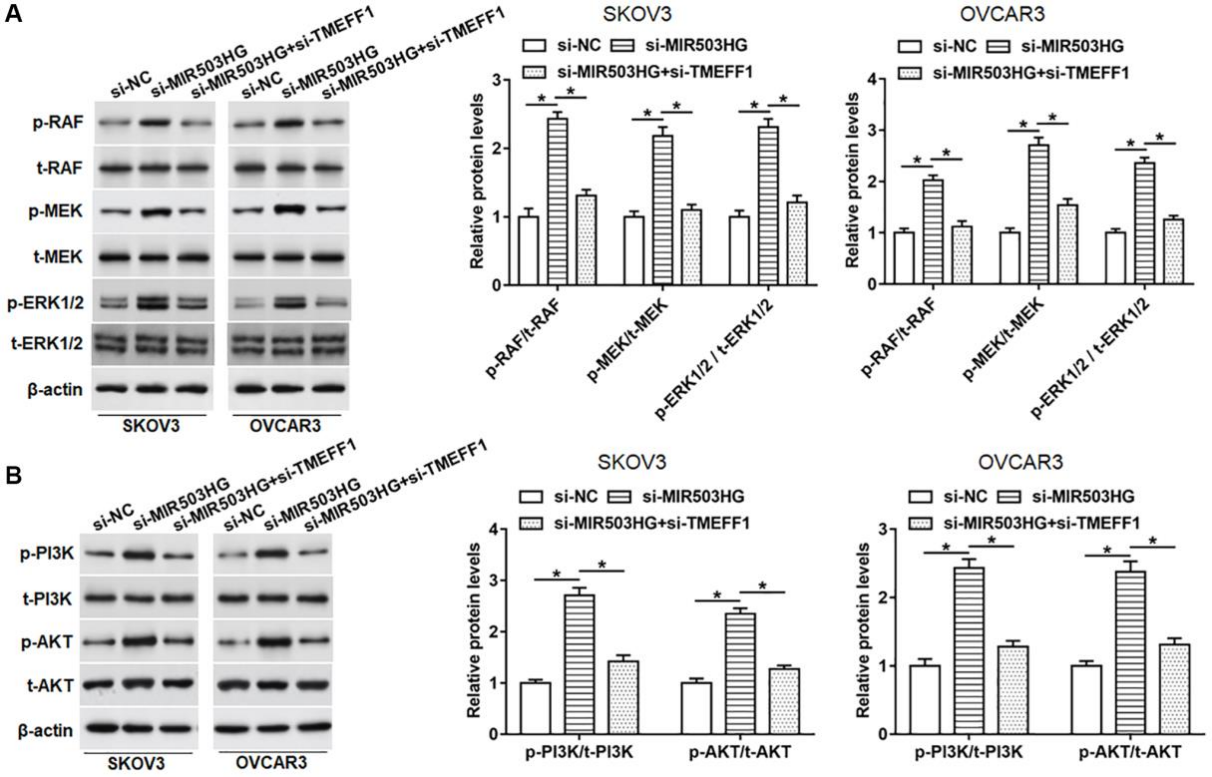
**Silencing MIR503HG activated MAPK and PI3K/AKT pathways by increasing TMEFF1 expression.** SKOV3 and OVCAR3 cells were transfected with MIR503HG siRNA alone or together with TMEFF1 siRNA, after transfection for 48 h, (**A**, **B**) the expression of phosphorylated RAF (p-RAF), p-MEK, p-ERK1/2, p-PI3K, and p-AKT, and total RAF (p-RAF), t-MEK, t-ERK1/2, t-PI3K, and t-AKT was assessed with Western blotting. ^*^P < 0.05. *n* = 6 in each group. Each test was repeated at least three times independently.

It is reported that TMEFF1 can promote the malignant behaviors of ovarian cancer cells by activating the MAPK and PI3K/AKT pathways. In order to explore whether MIR503HG affected the activities of MAPK and PI3K/AKT pathways through regulating TMEFF1, SKOV3 and OVCAR3 cells were transfected with MIR503HG siRNA alone or together with TMEFF1 siRNA. Our data revealed that the knockdown of MIR503HG obviously upregulated the protein levels of phosphorylated-RAF (p-RAF), p-MEK, p-ERK1/2, as well as p-PI3K and p-AKT, which was reversed by the science of TMEFF1 (Figure 6A, 6B). While the expression of total-Raf (t-Raf), t-MEK, t-ERK1/2, as well as t-PI3K and t-AKT did not change whether interference with MIR503HG or co-interference with MIR503HG and TMEFF1 (Figure 6A, 6B).

### Overexpression of MIR503HG impeded ovarian cancer xenograft tumor growth *in vivo*

**Figure 7:**
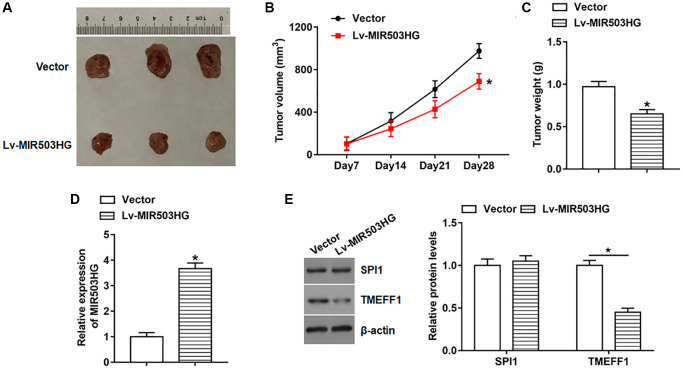
**Overexpression of MIR503HG impeded ovarian cancer xenograft tumor growth *in vivo*.** SKOV3 cells infected with lentiviral empty vector or Lv-MIR503HG were subcutaneously injected into the right flank of each mouse. Cell concentration was adjusted to 1 × 107 cells/mL, and each mouse was injected with 100 μL of cell suspension. (**A**) Representative images of tumors. (**B**) Tumor size was measured weekly with a vernier caliper. Four weeks later, mice were anesthetized, and (**C**) tumor weight was evaluated. (**D**, **E**) The expression of MIR503HG, SPI1, and TMEFF1 in tumor tissues was analyzed. ^*^P < 0.05. *n* = 10 in each group. Each test was repeated at least three times independently.

To explore the effect of MIR503HG on tumorigenesis, SKOV3 cells infected with lentiviral empty vector or Lv-MIR503HG were subcutaneously injected into the right flank of each nude mouse, and the tumor volume and weight were measured. The results revealed that overexpression of MIR503HG prominently hindered tumor growth (Figure 7A–7C). And the expression of MIR503HG was significantly upregulated and TMEFF1 protein level was observably declined in tumor tissues after MIR503HG overexpression (Figure 7D, 7E). While overexpression of MIR503HG in tumor tissues did not alter the protein level of SPI1 (Figure 7E).

## DISCUSSION

**Figure 8:**
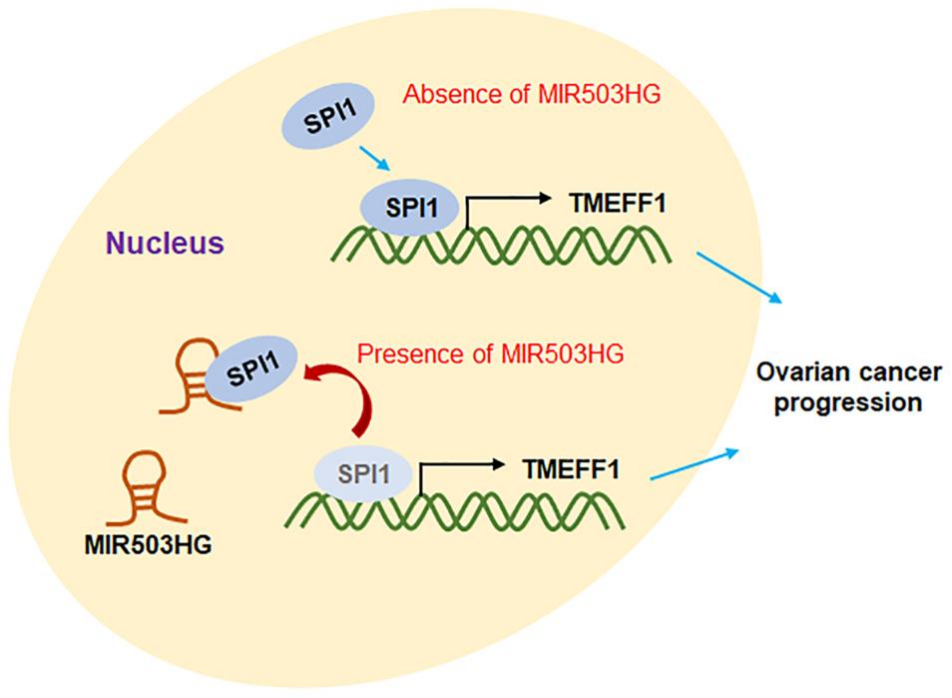
**Molecular mechanism diagram.** The transcription factor SPI1 binds to the TMEFF1 promoter region to promote the transcription of TMEFF1. Overexpression of MIR503HG can bind to SPI1, block the transcriptional activation of TMEFF1 by SPI1, and inhibit the expression level of TMEFF1, thereby hindering the progression of ovarian cancer.

As shown in Figure 8, MIR503HG inhibited SPI1-mediated transcriptional activation of TMEFF1 by binding to SPI1, and suppressed the expression of TMEFF1, thus hindering the progression of ovarian cancer.

lncRNAs are related to various types of gene regulation, including epigenetic regulation, transcription regulation or post-transcriptional regulation, which promote the development of many diseases including cancer [19]. Growing evidence indicated that MIR503HG hinders the progression of many tumors by regulating proliferation, apoptosis, invasion, EMT and metastasis. MIR503HG regulates the expression of Par4 through spongy miR-107 to inhibit colon cancer cell proliferation, migration and invasion, promote cell apoptosis, down-regulate N-cadherin and Vimentin, and up-regulate E-cadherin [20]. MIR503HG suppresses cell cycle arrest by decreasing cyclin D1 expression, thereby impeding the proliferation of non-small cell lung cancer cells [21]. By suppressing cell proliferation and invasion and promoting cell apoptosis *in vitro*, and hindering the growth of mouse xenograft tumors *in vivo*, MIR503HG exerts a tumor suppressor effect in cervical cancer [22]. In addition, MIR503HG may promote the methylation of miR-31-5p and act as a sponge to inhibit migration and invasion of ovarian cancer cells [23]. Our results indicated that MIR503HG expression was downregulated in collected ovarian cancer tissues and cell lines, and the ectopic expression of MIR503HG inhibited the proliferation, invasion and EMT progress, and promoted cell apoptosis in ovarian cancer cells. Moreover, overexpression of MIR503HG hindered the growth of xenograft tumors in nude mice, suggesting that MIR503HG played a tumor suppressor role in ovarian cancer. Whereas, interference with miR-503 did not change the effect of MIR503HG on cell proliferation, invasion and apoptosis, suggesting that MIR503HG functioned independently of miR-503.

Recent studies demonstrated that lncRNAs can regulate gene transcription by binding to transcription factors to play a carcinogenic or tumor suppressor effect in tumors. LINC00511 contributes to RXRA-mediated transcriptional activation of PLD1 as an oncogenic lncRNA in cervical cancer by recruiting the transcription factor RXRA [24]. LINC00982 can bind to the transcription factor HEY1 and inhibit its expression, and then promote the expression of CTSF by blocking the binding of HEY1 to the CTSF promoter, thus blocking the progression of gastric cancer [25]. Furthermore, SPI1 participates in tumor progression by playing a transcriptional regulatory role. LncRNA SNHG16 recruits the transcription factor SPI1 to promote the transcription of PARP9, thereby promoting the tumorigenicity of cervical cancer cells [13]. SPI1 acts as a lncRNA SNHG6 transcription factor to up-regulate SNHG6 expression and promote the migration and invasion of NSCLC cells [14]. Methyl-CpG binding protein 2 (MeCP2) helps SPI1 bind ZEB1 promoter to increase the transcription of ZEB1, thus maintaining the stemness and metastasis of colorectal cancer cells [26]. By using LncMAP database, we found that MIR503HG might interact with the transcription factor SPI1. And the subsequent RIP and RNA pull-down tests confirmed the binding between them. In order to explore which region of SPI1 bound to MIR503HG sequence, MIR503HG sequence was divided into five truncated parts according to the stem-loop structure of MIR503HG. And the RNA pull-down assay suggested that SPI1 mainly bound to MIR503HG fragment 21-134 nt. Interestingly, changes in the expression of MIR503HG did not affect the mRNA and protein levels of SPI1. Moreover, by using the ChIPBase database, we found that SPI1 might bind to the promoter region of TMEFF1. The luciferase reporter gene assay and ChIP assay showed that SPI1 directly bound to the TMEFF1 promoter region and the binding motif was “TCCCTACTTCCTCATCT”. Additionally, overexpression of MIR503HG prevented SPI1 from binding to the promoter of TMEFF1 and suppressed the promotion effect of SPI1 on the expression of TMEFF1, indicating that MIR503HG restrained TMEFF1 expression by competitively binding to SPI1.

The role of TMEFF1 in brain, endometrial and ovarian cancers has been investigated. The expression level of TMEFF1 in brain tumor tissues is lower than that in normal brain tissues, and its overexpression inhibits the growth of brain cancer cells [15]. TMEFF1 is highly expressed in endometrial cancer and is closely related to FIGO staging and lymph node metastasis. Interference with TMEFF1 inhibits the invasion, migration and EMT progress of endometrial cancer cells [27]. Butorphanol treatment suppressed the viability, migration, invasion and colony formation of ovarian cancer cells, and it was found that Butorphanol might play its role by decreasing TMEFF1 expression [28]. High TMEFF1 expression predicts shorter overall survival in ovarian cancer patients, and its ectopic expression *in vitro* promotes malignant progression of ovarian cancer cells [18]. The results in this study revealed that silencing TMEFF1 reversed the promotion effect of MIR503HG knockdown on cell proliferation, invasion, and EMT progress, as well as the inhibitory effect of MIR503HG knockdown on cell apoptosis, indicating that TMEFF1 acted as an oncogene in ovarian cancer, which was consistent with the previous reports.

The PI3K/AKT/ signaling pathway is one of the most important signaling pathways in cells. It regulates cell survival, growth, differentiation, metabolism and skeletal reorganization in a series of signal responses. Excessive activation of the PI3K signaling cascade is a common event in human cancers [29]. Mitogen-activated protein kinase (MAPK) pathway is a complex signal cascade that is often involved in tumorigenesis, tumor progression and drug resistance [30]. According to reports, TMEFF1 can promote the malignant behaviors of ovarian cancer and endometrial cancer cells by activating MAPK and PI3K/AKT signaling pathways [18, 27]. Our data demonstrated that interference with MIR503HG activated the MAPK and PI3K/AKT signaling pathways, while TMEFF1 silence rescued the effect of MIR503HG siRNA on the two pathways, suggesting that MIR503HG might regulate the MAPK and PI3K/AKT pathways by affecting the expression of TMEFF1, thereby participating in the progression of ovarian cancer.

In conclusion, MIR503HG expression was downregulated in ovarian tumor tissues and cells, and MIR503HG overexpression impaired the proliferative, invasive and EMT properties, and facilitated cell apoptosis in ovarian cancer cells. MIR503HG could bind to the transcription factor SPI1 to prevent SPI1 from binding to the TMEFF1 promoter region, thereby suppressing the tumorigenicity of ovarian cancer cells. Our findings may provide a promising therapeutic target for the intervention of ovarian cancer.

## MATERIALS AND METHODS

### Human tissue samples

Forty-five paired ovarian cancer tissues and adjacent normal tissues were obtained from ovarian cancer patients who underwent surgical resection between 2017 and 2019 at Baoji Maternal and Child Health Care Hospital. Inclusion criteria: 1) patients with epithelial ovarian cancer confirmed by pathological diagnosis; 2) patients voluntarily accept surgical treatment. Exclusion criteria: 1) patients with non-initial surgery; 2) patients with other primary tumors; 3) patients who received radiotherapy or chemotherapy before surgery; 4) patients suffering from other serious diseases; 5) patients with missing follow-up data. All tissues were immediately frozen in liquid nitrogen and stored at −80°C until use. This study was approved by the Ethics Committee of Baoji Maternal and Child Health Care Hospital. All patients signed informed consents. Clinical characterizations of ovarian cancer patients were shown in Table 1.

**Table 1:** The characteristics of ovarian cancer patients.

**Characteristics**	**Case (*n*, %)**
Age (years)	
≤50	26 (57.8%)
>50	19 (42.2%)
Pathologic type	
Serous	27 (60.0%)
Mucous	11 (24.4%)
Clear cell carcinoma	5 (11.1%)
endometrial	2 (4.4%)
Histologic grade	
G1-2	28 (62.2%)
G3	17 (37.8%)
FIGO stage	
I–II	15 (33.3%)
III–IV	30 (66.7%)
Lymph nodes metastasis	
No	38 (84.4%)
Yes	7 (15.6%)
Tumor size (cm)	
<4	20 (44.4%)
≥4	25 (55.6%)

Abbreviation: FIGO: Federation International of Gynecology and Obstetrics.

### Cell culture and transfection

Human ovarian cancer cell lines (OVCAR3, A2780, SKOV3, HeyA8), normal ovarian epithelial cell line (IOSE80), and HEK293T cells were obtained from the American Type Culture Collection (ATCC, Manassas, VA, USA). Cells were cultured in Dulbecco’s modified Eagle’s medium (DMEM; Gibco, Rockville, MD, USA) containing 10% fetal bovine serum (FBS; Gibco) at 37°C with 5% CO_2_.

Full-length sequence of MIR503HG and full-length DNA coding sequences of SPI1 and TMEFF1 were inserted into the pcDNA3.1 empty vector. MIR503HG small interfering RNA (siRNA), TMEFF1 siRNA, and negative control (NC) siRNA were synthesized by Invitrogen (Carlsbad, CA, USA). Lentiviral-mediated MIR503HG overexpression vector (Lv-MIR503HG) was obtained from Gene Pharma (Shanghai, China). Cell transfection was performed with Lipofectamine^®^ 3000 (Thermo, Waltham, MA, USA) according to the manufacturer’s instructions. Cells were infected with lentiviral empty vector or Lv-MIR503HG with a multiplicity of infection (MOI) of 100. The siRNA sequences that mentioned above were as follows: NC siRNA: 5′-UUCUCCGAACGUGUCACGUTT-3′; TMEFF1 siRNA: 5′-GCUCACUCAUGUU CUUAUUTT-3′; MIR503HG siRNA: 5′-CCACACUU CUUUGUUCCAATT-3′.

### Cell proliferation

Ovarian cancer cells were seeded into 96-well plates at a density of 2 × 10^3^ per well. Then, 10 μl of Cell counting assay kit-8 (CCK-8) solution (CCK8, Dojindo, Tokyo, Japan) was added into each well at the specified point in time, followed by incubation at 37°C for another 4 h. The absorbance at 450 nm was measured on a microplate reader (Sunrise; Tecan, Mannedorf, Switzerland).

### Cell invasion assay

Cell invasion was determined by using Transwell inserts (8.0-μm pore size; Costar, Cambridge, MA, USA). 500 μl of serum-free medium containing 2 × 10^4^ ovarian cancer cells were added into the upper chamber, which was pre-coated with Matrigel (BD Biosciences, San Jose, CA, USA). The bottom chamber was filled with 750 μl of DMEM medium containing 10% fetal bovine serum. After culturing for 24 h, cells in the upper chamber were carefully removed, and cells in the bottom chamber were stained with 0.1% crystal violet. The number of invaded cells was counted with a light microscope.

### Western blotting

Total protein was isolated from ovarian cancer cells and tissues by using RIPA lysis buffer (Beyotime Biotechnology, Nantong, Jiangsu, China) and determined with a BCA Protein Assay Kit (Thermo, Waltham, MA, USA). CelLytic^™^ NuCLEAR^™^ Extraction Kit (Sigma, St. Louis, MO, USA) was used to extract nuclear proteins. The same amount of proteins were separated with SDS-PAGE gel (10%), and then electro-transferred onto a PVDF membrane (Millipore, Massachusetts, USA). The membrane was blocked with 5% skimmed milk, followed by incubation with the following primary antibodies: anti-SPI1 (ab76543, 1:5000, Abcam), anti-TMEFF1 (ab133562, 1:3000, Abcam), anti-E-cadherin (#8834, 1:1000, Cell Signaling Technology), anti-N-cadherin (#4061, 1:1000, Cell Signaling Technology), anti-Vimentin (#49636, 1:1000, Cell Signaling Technology), anti-phosphorylated-RAF (p-RAF, #4431, 1:1000, Cell Signaling Technology), anti-p-MEK (ab96379, 1:3000, Abcam), anti-p-ERK1/2 (ab278538, 1:1000, Abcam), anti-p-PI3K (#4228, 1:1000, Cell Signaling Technology), anti-p-AKT (ab38449, 1:500, Abcam), anti-total-RAF (t-RAF, #4432, 1:1000, Cell Signaling Technology), anti-t-MEK (ab32576, 1:10000, Abcam), anti-t-ERK1/2 (ab17942, 1:1000, Abcam), anti-t-PI3K (#4255, 1:1000, Cell Signaling Technology), anti-t-AKT (ab18785; 1:1000, Abcam), and anti-β-actin (ab68183, 1:500, Abcam) at 4°C overnight. Next, the membrane was incubated with horseradish peroxidase-labeled secondary antibodies for 1 h at room temperature. The protein bands were detected using the ECL system (Thermo), and the integrated optical density was quantified with ImageJ software.

### Real time quantitative PCR (RT-qPCR)

Total RNA was isolated from ovarian cancer cells and tissues with TRIzol reagent (Invitrogen, Carlsbad, CA, USA) in accordance with the manufacturer’s protocol. 1 μg of RNA was reverse transcribed into cDNA by using a First Strand cDNA Synthesis kit (Takara, Dalian, China). RT-qPCR was carried out with the SYBR Green Master Mix (Takara) by using the following program: 95°C for 10 min, followed by 40 cycles of 95°C for 15 s, 60°C for 30 s, and 72°C for 15 s. Relative expression of candidate genes was calculated via the 2^−ΔΔCt^ method. GAPDH was used as the internal reference. Primer sequences were as follows: MIR503HG-Forward-5′-CAGCCTTCCTGAAAGAC CA-3′, Reverse-5′-TGTTGATGTAGTGTTCCTG GGT-3′; GAPDH-Forward-5′-CGCTCTCTGCTCCT CCTGTTC-3′, Reverse-5′-ATCCGTTGACTCCGA CCTTCAC-3′; SPI1-Forward-5′-GCGACCATTACT GGGACTTCC-3′, Reverse-5′-GGGTATCGAGGACG TGCAT-3′; TMEFF1-Forward-5′-GAGGGAGTCT GACGTAAGAGT-3′, Reverse-5′-AGTGTCCCC ATTTGATCCACA-3′.

### Dual luciferase reporter gene assay

The target genes of MIR503HG were analyzed using the LncMAP bioinformatics prediction website. The ChIPBase database was used to predict the binding site of SPI1 in the TMEFF1 promoter region. The wild-type and mutant-type promoter region of TMEFF1 were inserted into the pGL3-basic vector (Promega Corporation, Madison, WI, USA) to construct WT-TMEFF1 and MUT-TMEFF1 recombinant vectors. HEK293T cells were co-transfected with WT-TMEFF1/MUT-TMEFF1 and SPI1 overexpression vector, or co-transfected with WT-TMEFF1/MUT-TMEFF1, SPI1 overexpression vector and MIR503HG overexpression vector. After transfection for 48 h, the luciferase activity was detected with a dual-luciferase reporter gene assay analytical system (Promega) according to the manufacturer’s instructions.

### RNA immunoprecipitation (RIP) assay

The binding ability between MIR503HG and SPI1 was examined by using a RIP kit (Millipore, Billerica, MA, USA). Ovarian cancer cells were lysed in ice bath with RIPA lysate (P0013B, Beyotime Biotechnology, Shanghai, China) for 5 min. Next, the samples were centrifuged at 4°C for 10 min, and the supernatant was collected. Partial cell supernatant was used as input, and the remaining was incubated with magnetic beads coupled with anti-IgG or anti-SPI1 at 4°C overnight. Then, the beads were rinsed with washing buffer. The expression of MIR503HG was determined using RT-qPCR.

### RNA pull-down assay

RNA pull-down assay was performed as described previously [31]. Briefly, the RNAs were transcribed by using SP6/T7 RNA polymerase (Roche Diagnostics, Indianapolis, IN, USA) *in vitro*. Then, the transcribed RNAs were biotin-labeled by using Biotin RNA Labeling Mix (Roche), followed by treatment with RNase-free DNase I and purification with an RNeasy Mini Kit (Qiagen, Valencia, CA, USA). Ovarian cancer cells were collected and lysed with a protein lysis buffer, and then 1 mg of cell extract was mixed with 50 pmol of biotinylated RNA. 60 μl of Streptavidin agarose beads (Invitrogen, Carlsbad, CA, USA) was incubated with the samples for 1 h at room temperature. The beads were washed and boiled in SDS buffer, and the eluted proteins were measured with Western blotting.

### Chromatin immunoprecipitation (ChIP) assay

ChIP assay was performed to detect the binding of SPI1 to the promoter region of TMEFF1. In brief, ovarian cancer cells were cross-linked with 1% formaldehyde for 10 min, and the Sonicator was used to fragment chromatin to a length of 200–1000 bp. Next, the samples were incubated with anti-IgG or anti-SPI1 at 4°C overnight. The DNA-protein complex was harvest with Protein Agarose/Sepharose. 5 mmol/L NaCl was used for cross-linking to retrieve DNA. The enrichment of TMEFF1 promoter was determined by using RT-qPCR.

### Tumor xenograft in mice

A total of 20 female BALB/c nude mice (weighting 18–22 g, aged 4–6 weeks) were obtained from HuiAo Biotechnology (Beijing, China). Animals were fed at 25 ± 2°C and 45%–50% constant humidity, and had free access to clean water and food. To measure the tumorigenesis, SKOV3 cells infected with lentiviral empty vector or Lv-MIR503HG were subcutaneously injected into the right flank of each mouse. Cell concentration was adjusted to 1 × 10^7^ cells/mL, and each mouse was injected with 100 μL of cell suspension. Tumor size was measured weekly with a vernier caliper, and tumor volume was evaluated by using the formula: V = a × b^2^/2 (a: the longest diameter of the tumor; b: the shortest diameter of the tumor). Four weeks later, mice were anesthetized with 2% pentobarbital sodium (50 mg/kg) and sacrificed with cervical dislocation. Then, the tumor tissues were isolated and weighed.

### Statistical analysis

The data in this study were expressed as mean ± SEM. Data distribution normality and homogeneity of variance were assessed using the Shapiro-Wilk test and Levene test, respectively. One way analysis of variance (ANOVA) followed by Tukey HSD test were applied for evaluating the significance among multiple groups, and Student’s *t*-test was performed to assess the significance between two groups, according to the data normal distribution and homogeneity of variances. The value of *p* < 0.05 was considered statistically significant.

### Availability of data and materials

The datasets used during the present study are available from the corresponding author upon reasonable request.

### Ethical approval and consent to participate

This study was approved by the Ethics Committee of Baoji Maternal and Child Health Care Hospital (BJFY2021-026). All animal experiments were in accordance with the guide for the care and use of laboratory animals established by United States National Institutes of Health (Bethesda, MD, USA). All patients obtained informed consent before surgery. All human parts adhered to the ethical guidelines of the Declaration of Helsinki.

## Supplementary Materials

Supplementary Figure 1
