# Supplementary material for: MIR503HG impeded ovarian cancer progression by interacting with SPI1 and preventing TMEFF1 transcription
Source: Aging (Albany NY). 2022 Jun 28;14(13):5390–405. doi: 10.18632/aging.204147 (PMC9320548; doi:10.18632/aging.204147)
Supplement: Supplementary Figure 1 [file aging-14-204147-s001.pdf]

SUPPLEMENTARY FIGURE

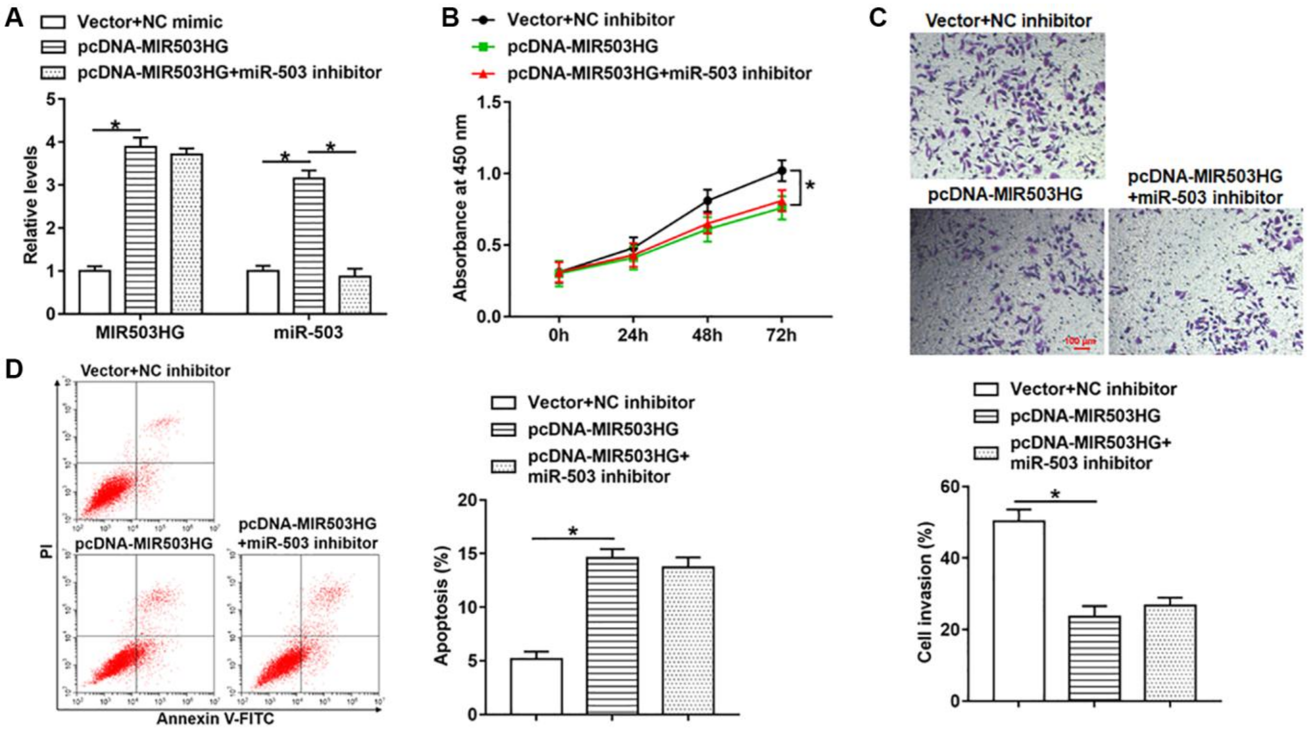

Supplementary Figure 1. Interference with miR-503 did not change the effect of MIR503HG on ovarian cancer cell behaviors. pcDNA-MIR503HG was transfected into SKOV3 and OVCAR3 cells alone or together with miR-503 inhibitor, after transfection for 48 h, (**A**) the expression of MIR503HG and miR-503 was detected. (**B**, **C**) CCK-8 and Transwell assays were carried out to analyze cell proliferation and invasion. (**D**) Flow cytometry was used to evaluate cell apoptosis. $^{*}P < 0.05$. $n = 6$ in each group. Each test was repeated at least three times independently.
